# Operation for Varicose Veins

**Published:** 1838-02-28

**Authors:** T. Harris

**Affiliations:** Surgeon to the hospital


### CLINICAL REPORTS.

### PENNSYLVANIA HOSPITAL.

*Operation for varicose veins, by* T. Harris, M. D. *Surgeon to the hospital.*

The subject was 66 years of age, and had suffered from leg ulcers for five years, which had been preceded for two years, by a varicose condition of the veins.

*Saturday, 3d February*, the man was introduced into the amphiteatre, and stood upon the table. A fold of skin over the saphena vein, where it passes over the knee-joint, was held up by an assistant, and a bistoury passed through, dividing the skin and cellular substance down to the vein. A needle, armed with a ligature, was passed under the vein, which was drawn out, and a piece of it, half an inch in length, removed. A firm compress was placed above and below the orifice, the edges of the wound drawn together by adhesive plaster, and a bandage applied from the toes up to above the knee. The man was put to bed, and the limb placed in a long fracture box. The bandage was not removed for a week. At the end of that time, the wound was lightly touched with nitrate of silver, and *Wednesday, 21st*, it had entirely healed. The veins are still visible through the skin, though considerably reduced in size. Bandage still applied.

